# Overexpression of E3 ubiquitin ligase Cbl attenuates endothelial dysfunction in diabetes mellitus by inhibiting the JAK2/STAT4 signaling and Runx3-mediated H3K4me3

**DOI:** 10.1186/s12967-021-03069-w

**Published:** 2021-11-19

**Authors:** Qingsong Jin, Liangyan Lin, Tiantian Zhao, Xiaoyan Yao, Yaqin Teng, Dongdong Zhang, Yongjun Jin, Meizi Yang

**Affiliations:** 1 Department of Endocrinology and Metabolism, Yantai Affiliated Hospital of Binzhou Medical University, No. 717, Mouping District, Yantai, 264100 Shandong Province People’s Republic of China; 2 Department of Pharmacology, School of Basic Medical Sciences, Binzhou Medical University, No. 522, Huanghe Third Road, Yantai, 264003 Shandong Province People’s Republic of China

**Keywords:** Diabetes mellitus, Cbl, Endothelial dysfunction, JAK2, STAT4, Human umbilical vein endothelial cells

## Abstract

**Background:**

Diabetes mellitus (DM), a most common chronic disease, is featured with impaired endothelial function and bioavailability of nitric oxide (NO), while E3 ubiquitin ligase appears to alleviate endothelial dysfunction as a promising option for DM treatment. Herein, we aimed to determine whether E3 ubiquitin ligase casitas B-lineage lymphoma (Cbl) alleviates endothelial dysfunction in DM rats by JAK2/STAT4 pathway.

**Methods:**

A rat model of DM was developed through intraperitoneal injection of streptozotocin, followed by collection of aortic tissues to determine the expression of Cbl, JAK2, runt-related transcription factor 3 (Runx3) and STAT4. Human umbilical vein endothelial cells (HUVECs) were cultured in high glucose (HG) condition to induce DM as an in vitro model. With gain- and loss-function method, we assessed the aberrantly expressed Cb1 on endothelial dysfunction, NO production and apoptosis of HUVECs.

**Results:**

Cbl was reduced in DM rat tissues and HG-induced HUVECs, where JAK2, Runx3 and STAT4 were elevated. It was found that overexpression of Cbl alleviated endothelial dysfunction by increasing NO production and restoring vasodilation and suppressing apoptosis of HUVECs. Mechanistically, Cb1 enhanced JAK2 ubiquitination and decreased JAK2 and STAT4 expression, where STAT4 improved Runx3 expression by regulating histone H3 lysine 4 trimethylation level. Overexpression of JAK2 and STAT4, or Runx3 increased apoptosis of HUVECs, abrogating the effect of Cb1 on endothelial function.

**Conclusion:**

In conclusion, Cbl alleviates endothelial dysfunction by inactivation of the JAK2/STAT4 pathway and inhibition of Runx3 expression in DM. These evidence might underlie novel Cbl-based treatment against DM in the future.

**Supplementary Information:**

The online version contains supplementary material available at 10.1186/s12967-021-03069-w.

## Background

Diabetes mellitus (DM) has become the most challenging public health problem of the twenty-first century, imposing endless burden both globally and nationally; however, it is estimated by International Diabetes Federation that the amount of patients with DM will rise to 642 million by 2040 [1]. DM is characterized by rapid glucose increase since the feedback loops between insulin action and insulin secretion do not function properly, but there is no immediate prospect of a cure and lifelong management is required [2]. Over the past two decades, biomarkers from the pathways of subclinical inflammation and endothelial dysfunction have improved our understanding of DM pathophysiology yet the ability of these biomarkers remains debatable [3]. Biomarkers for DM deserve more investigation and also require further studies since they hold the potential of easing the burden imposed by DM.

In the context of DM, endothelial function is damaged and bioavailability of nitric oxide (NO) is reduced in endothelial progenitor cells (EPCs) [4]. EPCs are essential to blood vessel formation and repair of damaged endothelium; in DM, the circulating EPC count is low and their functionality is impaired [5]. The reduction of NO bioavailability in EPCs is inversely correlated with patient’s plasma glucose [4]. DM impairs endothelial NO synthase activity, thus leading to diminished NO bioavailability and the consequent pro-atherogenetic alterations [6]. Endothelial dysfunction results in metabolic disturbance, which in turn exacerbates endothelial and vascular dysfunction, and contributes to other pathological conditions, where NO is a major player in endothelial function [7]. Endothelial NO deficiency or endothelial dysfunction contributes to advanced nephropathy and retinopathy in patients with diabetes [8]. Also, vasodilation, induced by insulin through NO-dependent mechanisms in skeletal muscle, contributes to both insulin sensitivity and responsiveness [7]. Though several circulating biomarkers have been proposed as indicators of endothelial dysfunction, such as tumor necrosis factors, up to date, the association between DM and endothelial dysfunction requires investigation [9]. Notably, high glucose (HG) culture allows human aortic endothelial cells to downregulate ubiquitin ligase Casitas B-lineage lymphoma (Cbl) expression [10]. Cbl was revealed as a significantly poorly-expressed gene in type II DM from bioinformatics analysis prior to our investigation. As a member of E3 ubiquitin ligase, Cbl negatively regulates the receptor tyrosine kinase signaling by ubiquitination and natural killer cell function [11]. Cbl-b modulates innate immune responses, and contributes to host defense to pathogens and anti-tumor immunity [12]. Importantly, Cbl-b prevents insulin resistance by inactivation of adipose tissue macrophages, as it negatively regulates signals associated with migration and activation [13]. Cbl-b is implicated to regulate T cell activation threshold and protect against DM, while Cbl-b deficiency predisposes to diabetes and formation of islet autoantibodies in mice [14]. Of note, Cbl has been found to reduce janus tyrosine kinase 2 (JAK2) stability and signal transduction in human hematopoietic stem/progenitor cells while Cbl knockout decreases JAK2 degradation yet increases JAK2 protein and signal transduction [15]. More importantly, phosphorylated JAK2 (p-JAK2) expression has been found to be upregulated in DM rats where JAK2 contributes to diabetic neuropathy [16], while HG environment also induces the activation of signal transducer and activator of transcription 4 (STAT4), facilitating the recruitment of signal transducer [17]. Also, p-JAK level has been detected to be diminished by knocking out Cbl in activated 293 T cells [18]. JAK2 activation has been demonstrated to be enhanced by Cbl-mediated ubiquitination of JAK2 with an ubiquitination site identified important for promoting JAK2 activation by Cbl [18]. In addition, STAT4 has been reported to upregulate the histone H3 lysine 4 trimethylation (H3K4me3) modification level of the runt-related transcription factor 3 (Runx3) promoter region to activate the transcription of the Runx3 gene in activated natural killer cells [19]. Moreover, Runx3 holds the potential of aggravating endothelial dysfunction in DM [20].

Given the aforementioned evidence, it was inferred that Cbl might alleviate endothelial dysfunction in DM by promoting NO production and inhibiting human umbilical vein endothelial cell (HUVEC) apoptosis through downregulation of Runx3 via the inactivated JAK2/STAT4 signaling pathway. Herein, we established a rat model of DM and HG-induced HUVECs model with vasodilation and NO content as two of important indexes.

## Materials and methods

### Ethical statement

This experiment was approved by the Ethics Committee of Yantai Affiliated Hospital of Binzhou Medical University and conducted in compliance with the *Declaration of Helsinki*. All patient individuals provided informed written consent documents. The experiments involving animals followed the recommendations in the Guide for the Care and Use of Laboratory Animals of the National Institutes of Health.

### Bioinformatics analysis

DM-related microarray dataset GSE26168 [21], including 15 blood samples for control and 9 blood samples of DM and GSE29221 [22], including 12 normal skeletal muscle samples and 12 skeletal muscle samples of DM were retrieved from Gene Expression Omnibus database (https://www.ncbi.nlm.nih.gov/geo/). The mRNA dataset (GSE26168) was analyzed by using the platform GPL6883 (Illumina HumanRef-8 v3.0 expression beadchip). The mRNA dataset (GSE29221) was analyzed by using the platform GPL6947 (Illumina HumanHT-12 V3.0 expression beadchip). The downloaded raw data was analyzed by the Affy R package and the limma R package was applied to identify differentially expressed genes (DEGs) with |logFoldChange|> 1 and adjusted *p* value < 0.05 as the threshold value. The adjusted *p* values were used to reduce the false positive rate through Benjamini and Hochberg false discovery rate method by default. A heat map displaying DEGs was plotted using “pheatmap” package (https://cran.r-project.org/web/packages/pheatmap/index.html). An intersection of DM-related genes was obtained by means of jvenn (http://jvenn.toulouse.inra.fr/app/example.html). E3 ligase of critical factors was predicted on Ubibrowser database (http://ubibrowser.ncpsb.org/ubibrowser/home/index). Protein–protein interaction (PPI) network of related genes was plotted through GeneMANIA (http://genemania.org/search/).

### Mouse model of DM

Male Sprague–Dawley rats (4–8 weeks old, 180–220 g) obtained from Charles River Company (101, Beijing, China, buy.vitalriver.com) were maintained under specific pathogen free condition, with temperature of 24–26 °C, humidity of 45–55% and free access to food and water.

The rats fasted for 16 h and then administered with phosphate buffered saline (PBS) or 60 mg/kg Streptozotocin (STZ) in 0.1 mmol/L citric acid buffer solution (pH = 4.5, 1%) through intraperitoneal injection. After 72 h, rat glucose level was detected with glucometer. When glucose stabled for 7 days, rats with the fasting blood glucose over 16.7 mmol/L were considered diabetic [23]. Then, rats were weighted and blood samples were collected to determine blood glucose, followed by lentivirus infection in vivo. Briefly, 50 μL of lentivirus (5 × 10^7^ TU/mL) overexpressing Cbl (oe-Cbl), oe-Runx3, and empty vectors for negative control (NC) (oe-NC; Shanghai Genechem Co., Ltd., Shanghai, China) were directly injected into DM rat aorta (n = 6) [24]. Six normal rats were taken as controls (Con) and 6 DM rats did not accept injection. After 2-week feeding, all rats were euthanized for subsequent analysis.

### Hematoxylin–eosin (HE) staining

In brief, aortic tissues were fixed, dehydrated and embedded in paraffin. The paraffin-embedded sections were baked, dewaxed with xylene, dehydrated with ethanol of gradient concentrations, and stained with hematoxylin (Beyotime Biotechnology Co., Ltd., Shanghai, China) for 5 min and with eosin (Beyotime) for 2 min. Then, the sections were dewaxed with xylene, dehydrated with ethanol of gradient concentrations, sealed with neutral balsam, and observed under a microscope (DMI3000, Leica, Wetzlar, Germany).

### Measurement of vasodilation

Blood vasodilation was measured by MPA polygraph (Alcott Biotech Co., Ltd., Shanghai, China). Rat aorta was placed in precooling oxygen-saturated K-H solution (Sinopharm Chemical, China) to remove adjacent connective tissues and then cut into rings (3–4 mm in length). The ring was hung beyond 10 mL of K-H solution, and continuously bubbled with carbogen (95% O_2_ and 5% CO_2_) at 37 °C. With MPA polygraph adjusted to base-line levels, aortic rings were placed in the middle of bath and connected to MPA. After pre-equilibration the rings were stimulated with norepinephrine (3 × 10^–7^ mol/L) and the vasodilation was restored to base-line, with K-H solution refreshed every 15 min. Following contraction with phenylephrine (PHE, 1 × 10^–6^ mol/L), the rings in chamber A and B were exposed to acetylcholine (ACH, 10^–8^ to 10^–4^ mol/L) or sodium nitroprusside (SNP, 10^–9^ to 10^–5^ mol/L), respectively, to assess vasodilation. The relaxation responses to ACH and SNP were calculated as a percentage of the response to PHE.

### Immunohistochemistry (IHC)

The paraffin-embedded rat aortic tissues were sliced and the sections were routinely dehydrated with gradient ethanol and next immersed in potassium citrate at 90 °C for 10 min followed by antigen retrieval. The sections were washed with PBS three times and next added with 3% H_2_O_2_ to inactivate endogenous peroxidase for 10 min. After blocked with goat serum (Solarbio, Beijing, China) for 20 min, the sections were incubated with anti-rabbit Cbl (1: 200, PA5-8292, Invitrogen, Carlsbad, CA, USA), anti-rabbit p-JAK2 (1: 2000, ab32101, Abcam, UK), anti-rabbit p-STAT4 (1: 100, ab28815, Abcam), and anti-mouse Runx3 (1: 500, ab135248, Abcam) overnight at 4 °C. The samples were then incubated with secondary goat anti-mouse immunoglobulin G (IgG) (ab150113, Abcam) or goat anti-rabbit IgG (ab150077, Abcam) for 1 h at room temperature. Finally, the sections were developed with diaminobenzidine (DAB; ZLI-9017, ZSGB-BIO, Beijing, China) and 4 sections of each sample were observed under microscope (DMI3000, Leica Biosystems, Shanghai, China) in three random fields, as positive cell percentage was calculated.

### Isolation of HUVECs

A newborn umbilical cord was taken from healthy pregnant women, placed in normal saline, and fixed by a rubber tube. The cord was repeatedly washed with normal saline to clear off blood. Then, the umbilical cord was filled with 15 mL 0.5% trypsin (Gibco, Carlsbad, CA, USA) as both ends were clamped with hemostatic forceps in a 37 °C water bath for 20 min. Then the solution was discharged to a centrifuge tube, and 20% calf serum-containing 199 medium (Gibco) was added to stop the reaction. Following 2 washes with D-HANKS solution (Solarbio), the mixture was centrifuged for 10 min with the supernatant removed. Then, the cells were washed twice with D-HANKS solution again, suspended in 199 culture medium containing 20% fetal bovine serum (FBS), and seeded onto a culture flask for culture. Next day, the medium was changed, and then renewed every 3 days. When the flask was covered with cells, medium was changed to serum-free culture medium for another 48-h culture, after which the cells could be used for subsequent experiments.

### Cell culture and infection

Primary HUVECs were cultured in Dulbecco’s Modified Eagle Medium (DMEM; Gibco) containing 10% FBS (Gibco) or HG medium (Gibco) at 37 °C with 5% CO_2_. Then, the HUVECs were seeded into 6-well plates with 2 mL DMEM for 1-day culture in an incubator. When confluence reached 80%, HUVECs from DMEM (Con) or HG medium were respectively infected with lentivirus expressing oe-Cbl, oe-Runx3, oe-STAT4, oe-JAK2, short hairpin RNA (shRNA) against STAT4 (sh-STAT4) (#1, #2), sh-Cbl(#1, #2), sh-NC and oe-NC (Genechem) according to the instructions (Invitrogen) with virus titer multiplicity of infection = 5.

### Western blot analysis

Proteins were extracted from the aorta tissues or the HUVECs cultured in normal medium or HG medium using phenylmethylsulfonylfluoride-contained radioimmunoprecipitation assay lysis buffer (Beyotime). After determination of the protein concentration by bicinchoninic acid kit, 30 μg of protein samples were then separated by 10% sodium dodecyl sulfate polyacrylamide gel electrophoresis (SDS-PAGE) and transferred onto polyvinylidene fluoride membranes (Amersham, Chicago, Illinois, USA). After blocked with skim milk powder for 1 h, the membranes were incubated with primary antibodies at 4 °C overnight and then with horseradish peroxidase-labeled goat anti-rabbit or goat anti-mouse secondary antibodies (1: 10,000, Jackson, West Grove, PA, USA) for 1 h. Subsequently, the membranes were developed and luminous intensity was detected by optical luminometer (GE Healthcare, Little Chalfont, Buckinghamshire, UK). Image J analysis software (Media Cybernetics, USA) was applied to quantify band intensity with β-actin as reference. The primary antibodies used in the experiment included: anti-rabbit Cbl (1: 2000, ab32027, Abcam), anti-rabbit endothelial NO synthase (eNOS) (1: 1000, ab76198, Abcam), anti-rabbit JAK2 (1: 5000, ab108596, Abcam), anti-rabbit p-JAK2 (1: 2000, ab32101, Abcam), anti-rabbit STAT4 (1: 2000, ab235946, Abcam), anti-rabbit p-STAT4 (1: 1000, ab28815, Abcam), anti-mouse Runx3 (1: 2000, ab135248, Abcam), and anti-mouse β-actin (1: 5000, ab8225, Abcam).

### Reverse transcription quantitative polymerase chain reaction (RT-qPCR)

Total RNA was extracted from HUVECs using Trizol Reagent (15,596,026, Invitrogen) and reversely transcribed into cDNA with PrimeScript RT reagent Kit (RR047A, TaKaRa, Toyko, Japan), with RNA concentration measured by Nanodrop 2000 (Thermo Fisher Scientific; Waltham, MA, USA). RT-qRCR was performed using SYBR® Premix Ex TaqTM (Tli RNaseH Plus) kit and ABI PRISM® 7500 system (Thermo Fisher Scientific). Transcriptional level of genes was calculated by 2 − ^ΔΔCt^ quantification method with β-actin as internal reference. The primers (GenePharma) are shown in Additional file 1: Table S1.

### Ubiquitination assay

After transfection for 48 h, HUVECs were treated with 20 μmol/L protease inhibitor MG-132 for 6 h and subjected to immunoprecipitation (IP) using the IP kit from Thermo Fisher Scientific. HUVECs were lysed with IP lysis buffer (protease inhibitor) for 5 min and next centrifuged at 13,000 rpm for 10 min at 4 °C with the supernatant collected. Cell lysate was treated with agarose resin and incubated with antibody to JAK2 at 4 °C overnight. Then, the mixture was interacted with A/G Agarose (Pierce, Rockford, IL, USA) for 2 h and centrifuged at 1000 × g for 1 min. The resin was washed three times with IP lysis/washing buffer and eluted twice with conditional buffer. Then, the sample was detected by Western blot analysis with anti-rabbit ubiquitin (ab134953, 1: 1000, Abcam).

### Half-life assay

HUVECs were detached with 0.25% trypsin and suspended in DMEM containing 10% serum to obtain the single cell suspension. Then, the cells were seeded onto a 6-well plate for 24-h culture, treated with 40 mg/mL cycloheximide (CHX) for 0, 1, 2, and 4 h, followed by separation through SDS-PAGE. Protein half-life was analyzed by Western blot analysis to detect JAK2 expression.

### NO detection

NO production in rat serum or HUVECs cultured in normal medium or HG medium was measured using NO detection kit, according to Griess method. Rat blood was centrifuged for 10 min at 4 °C and the supernatant was taken. The specimens and the diluted standard sample were added to 96-well plates, followed by addition of 50 μL Griess Reagent I and Griess Reagent II to each well. The absorbance at 540 nm was detected by a microplate reader (BioTek, VT, USA) and normalized to concentration of NO.

### Flow cytometry

HUVECs were stained with Annexin-V-fluorescein isothiocyanate (FITC) apoptosis kit (K201-100, Biovision, Milpitas, CA, USA) and centrifuged with culture medium removed. Then, the cells were incubated with 5 μL Annexin-V-FITC (green fluorescence) for 15 min and stained with propidium iodide solution (red fluorescence) for 5 min. HUVECs were detected by flow cytometer (BD Biosciences, Franklin Lakes, NJ, USA) with absorbance measured at 488 mm and the result was analyzed by Flow Jo 7.0 Analyzer (Becton, Dickinson and Company, NJ, USA).

### Chromatin immunoprecipitation (ChIP)-qPCR

ChIP was performed according to the instructions of EZ ChIP Kit (Millipore, Billerica, MA, USA). HUVECs from normal medium and rat aortic tissues were fixed with 1% formaldehyde at room temperature for 10 min for crosslinking. HUVECs were resolved in SDS Lysis Buffer + Proteinase Inhibitor Cocktail II (10^6^ cells/100 μL SDS solution). Afterward, cell precipitate was sonicated to DNA fragment at 200—1000 bp. The fragment was then probed with normal IgG antibody (Millipore), anti-rabbit STAT4 polyclonal antibody (5 μg, 71–4500, Invitrogen) and anti-mouse H3K4me3 monoclonal antibody (5 μg, ab185637, Abcam) for ChIP. Then, the purified DNA product was detected by RT-qPCR to determine whether the DNA sequence and relative content of the target gene were present in the antibody-complex. With Input as internal reference, gene expression was calculated by 2 − ^ΔΔCt^ quantification. The sequences of Runx3 are listed in Additional file 1: Table S2.

### Terminal deoxynucleotidyl transferase (TdT)-mediated 2'-deoxyuridine 5'-triphosphate nick end-labeling (TUNEL) staining

The paraffin-embedded tissues were cut into sections, whilst sections were dewaxed in xylene and hydrated with ethanol of gradient concentrations. Sections were immersed in 3% H_2_O_2_ for 10 min, washed in PBS for 5 min, and treated with 50 μL 20 μg/mL proteinase K (Sigma, St Louis, MO, USA) for 20 min at room temperature to remove tissue protein. Then, the sections were incubated with 50 μL TdT reaction solution, away from light at 37 °C for 1 h, while reaction solution without TdT was taken as a negative control, followed by incubation with 50 μL peroxidase-labeled anti-digoxigenin for 30 min in the dark. Finally, the sections were developed with DAB for 10 min, counterstained with hematoxylin, and observed under an optical microscope (Nikon, Tokyo, Japan) in five randomly-selected fields, while the apoptotic ratio was calculated as positive staining cells/all cells × 100%.

### Statistical analysis

The data were processed using SPSS 21.0 statistical software (IBM Inc., Armonk, NY, USA). Measurement data were expressed as mean ± standard deviation. The data between two groups was analyzed by unpaired *t*-test. The data among multiple groups were analyzed by one-way analysis of variance (ANOVA) with Tukey’s post hoc test. Data at different time points among groups were compared by two-way ANOVA, followed by Tukey’s post hoc test. * *p* < 0.05 was considered statistically significant.

## Results

###  Cbl is poorly expressed in aortic tissues of DM rats and HG-induced cell model

**Fig. 1:**
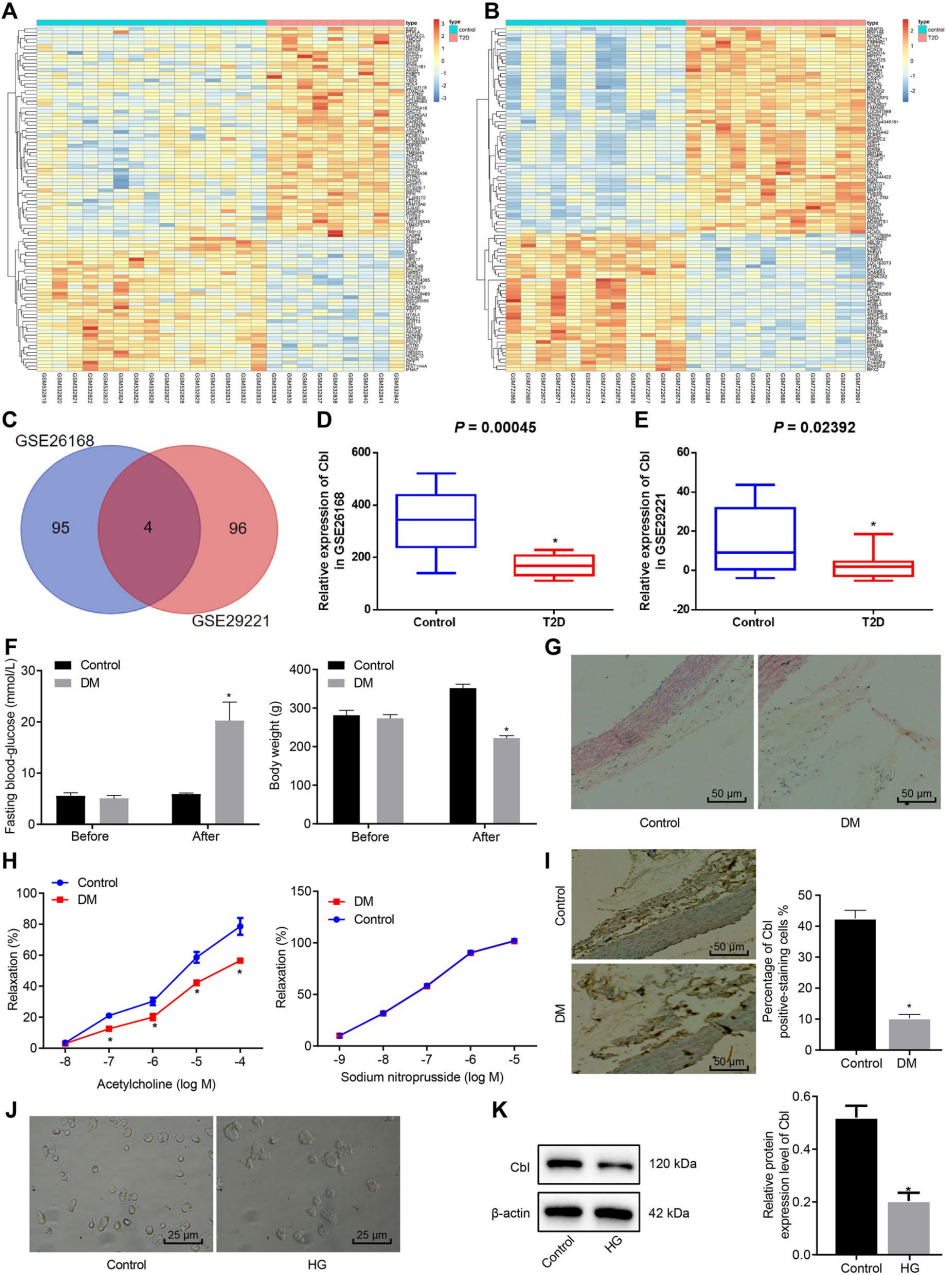
Differential expression of Cbl in the microarray data, DM rat model, and HG-induced HUVEC model. **A** The downregulated genes in DM retrieved from GSE26168 microarray. **B** The downregulated genes in DM retrieved from GSE29221 microarray. **C** The intersection of top 100 significantly downregulated genes in the GSE26168 and GSE29221 datasets by Venn diagram; the two circles in the graph represent the downregulated genes in the two datasets respectively, and the overlapped part represents the intersection of two groups of data. **D** The boxplot of Cbl expression in GSE26168 dataset; the left blue box represents the normal samples, and the right red box represents the type 2 DM samples. **E** The boxplot of Cbl expression in GSE29221 dataset; the left blue box represents the normal samples, and the right red box represents the type 2 DM sample. **F** Quantification of fasting blood glucose and body weight of rat before and after modeling. **G** HE staining of aortic tissues in DM rats or control rats (× 200). **H** Quantification of the aortic relaxation response to ACH and SNP in DM rats or control rats. **I** IHC assay of positive-Cbl protein in DM rat aortic tissues and corresponding quantification (× 200); n = 6. **J** Morphology observation of HUVECs under Con or HG condition (× 400). **K** Western blot analysis of Cbl expression of HUVECs cells under Con or HG condition. * *p* < 0.05 *vs.* the Con group. Data were measurement data, and presented as mean ± standard deviation. The data between two groups was analyzed by unpaired *t*-test. Data at different time points among groups were compared by two-way ANOVA, followed by Tukey’s post hoc test. Cell experiment was repeated three times independently

At the initial stage of our investigation, we analyzed the DEGs in GSE26168 and GSE29221 datasets by R language (Fig. 1A, B). Then, the intersection of the top 100 downregulated genes in the two datasets was selected by *p* value, and four genes (ANGPTL2, CHST7, PRRX2, and Cbl) were obtained as illustrated in the Venn map (Fig. 1C). Furthermore, the Cbl expression data in the GSE26168 and GSE29221 datasets were obtained to draw a boxplot, with the significant difference calculated by *t*-test. It was found that Cbl was significantly poorly expressed in type 2 DM (Fig. 1D, E).

Subsequently, an STZ-induced diabetic rat model was established where we analyzed the expression level of Cbl and measured vasodilation in the aortic tissues of DM rats. As revealed in Fig. 1F, the blood glucose dramatically elevated and weight of DM rats decreased, indicating successful establishment of DM rat models. After two weeks, rats were euthanized and dissected for observation. The results of HE staining (Fig. 1G) revealed the smooth aorta intima, intact endothelial cells, and neatly arranged smooth muscle cells in the media layer in Con rats while DM rats lost integrity of aorta intima, exhibiting increased volume of endothelial cells with partial shedding, and disordered smooth muscle cells. Additionally, accumulation of ACH concentration decreased endothelial-dependent vasodilation in a dose-dependent manner in DM rats (*p* < 0.05), but alteration of SNP hardly affected the capacity of vasodilation (*p* > 0.05) (Fig. 1H), indicating that endothelium-dependent vasodilation rather than nonendothelium-dependent vasodilation was alleviated in rats with DM. Furthermore, IHC noted that Cbl expression rallied on cytoplasm and its expression level was decreased in aorta tissues of DM rats relative to controls (Fig. 1I). To confirm the aberrant expression of Cbl, we exposed primary HUVECs to HG medium to mimic DM in a cell model. Under the microscope, we confirmed the establishment of HG-induced cell model based on the fact that HG culture changed the morphology of HUVECs from oval shape to spindle shape (Fig. 1J). We found that Cbl expression decreased in the HUVECs exposed to HG (Fig. 1K). Taken altogether, rats with DM had decreased aorta endothelium-dependent vasodilation with declined expression of Cbl, and HG-treated HUVECs also had lower Cbl expression.

### Cbl inhibits the JAK2/STAT4 pathway and affects endothelial cell apoptosis

**Fig. 2:**
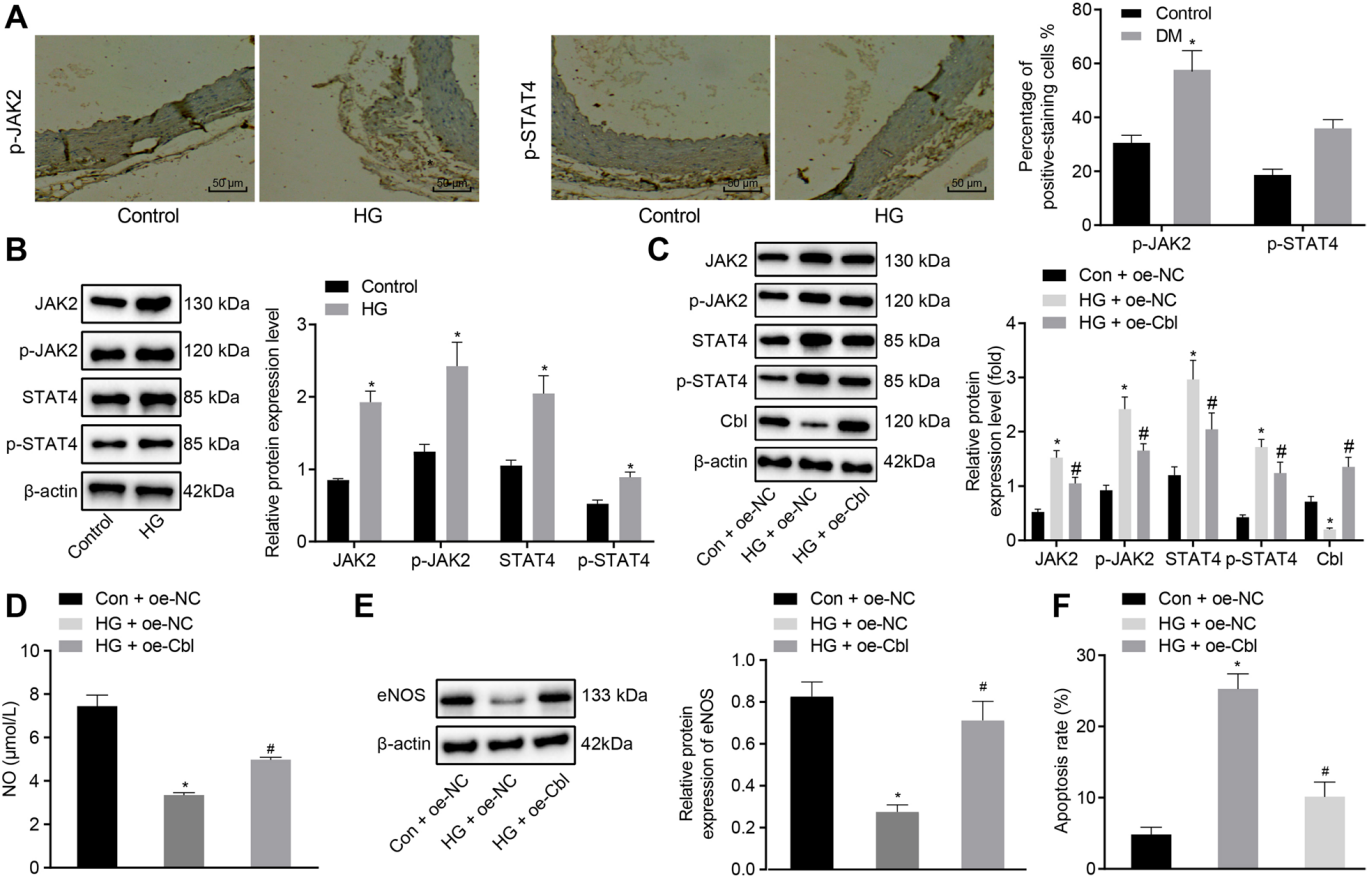
Effects of Cbl-mediated JAK2/STAT4 pathway on apoptosis of HUVECs. **A** Positive rate of p-JAK2 and p-STAT4 protein in rat aortic tissues from Con and DM rats (× 200). * *p* < 0.05 *vs*. the Con group, n = 6. **B** Western blot analysis of Cbl expression in HUVECs under Con or HG condition. * *p* < 0.05 *vs.* the Con group. **C** Western blot analysis of Cbl, JAK2, p-JAK2, p-STAT4 and STAT4 expression in Con or HG-treated HUVECs upon treatment with oe-NC or oe-Cbl. * *p* < 0.05 *vs.* the Con + oe-NC group and # *p* < 0.05 *vs*. the Con + oe-Cbl group. **D** Quantification of NO level in the culture supernatant of HUVECs upon treatment with Con + oe-NC, HG + oe-NC, and HG + oe-Cbl detected by Griess assay. * *p* < 0.05 *vs.* the Con + oe-NC group and # *p* < 0.05 *vs*. the Con + oe-Cbl group. **E** eNOS protein level in HG-induced HUVECs determined by Western blot analysis. **F** Flow cytometry of apoptosis of HUVECs and corresponding quantification upon treatment with Con + oe-NC, HG + oe-NC, and HG + oe-Cbl. * *p* < 0.05 *vs.* the Con + oe-NC group and # *p* < 0.05 *vs*. the HG + oe-NC group. Data were measurement data, and presented as mean ± standard deviation. The data between two groups was analyzed by unpaired *t*-test. Data among groups were analyzed by one-way ANOVA, followed by Tukey’s post hoc test. Cell experiment was repeated three times independently

Cbl in hematopoietic stem/progenitor cells was found to down-regulate the stability and signal transduction of JAK2 [15]. With aforementioned evidence indicating the regulatory action of Cbl on the JAK2 signaling pathway, we then attempted to investigate whether the mechanism is functional in HUVECs. IHC was performed to detect the expression of p-JAK2 and p-STAT4 on the aortic tissues of DM rats. It was evident that p-JAK2 and p-STAT4 were both expressed in the nucleus and cytoplasm where their expression in the aorta tissues were significantly upregulated in DM rats (Fig. 2A). We also confirmed the upregulated expression of JAK2, p-JAK2, STAT4, and p-STAT4 in HUVECs under HG culture by Western blot analysis (Fig. 2B). Then, we cultured primary HUVECs in HG medium and infected them with specific lentivirus harboring overexpressed/silenced Cbl. According to Western blot analysis, HG culture decreased the expression of Cbl in HUVECs but increased the expressions of JAK2, p-JAK2, p-STAT4 and STAT4; treatment of oe-Cbl resulted in an increase of Cbl expression in HUVECs but decreased expression of JAK2, p-JAK2, p-STAT4 and STAT4, suggesting that Cbl might inhibit the JAK2/STAT4 pathway.

NO is a signal molecule that maintains the physiological functions of endothelial cells and it can regulate vascular tension through biological properties [6], which is related to the endothelium-dependent relaxation function. We found that compared with control treatment, HG exposure decreased the content of NO in the culture supernatant of HUVECs (Fig. 2D) but additional overexpression of Cbl induced the production of NO in the supernatant of HUVECs. Further Western blot analysis on eNOS protein level determination in HG-induced HUVECs revealed downregulation of eNOS, which was reversed by overexpressed Cbl (Fig. 2E). Flow cytometry was conducted to assess the effect of Cbl on HUVECs and revealed that apoptosis was increased in HG-treated HUVECs yet decreased in the presence of oe-Cbl (Fig. 2F). These above evidence elucidate that overexpression of Cbl might inhibit the activation of the JAK2/STAT4 pathway and reduce apoptosis caused by HG in HUVECs.

### Cbl inhibits the JAK2/STAT4 pathway through promoting JAK2 protein ubiquitination

**Fig. 3:**
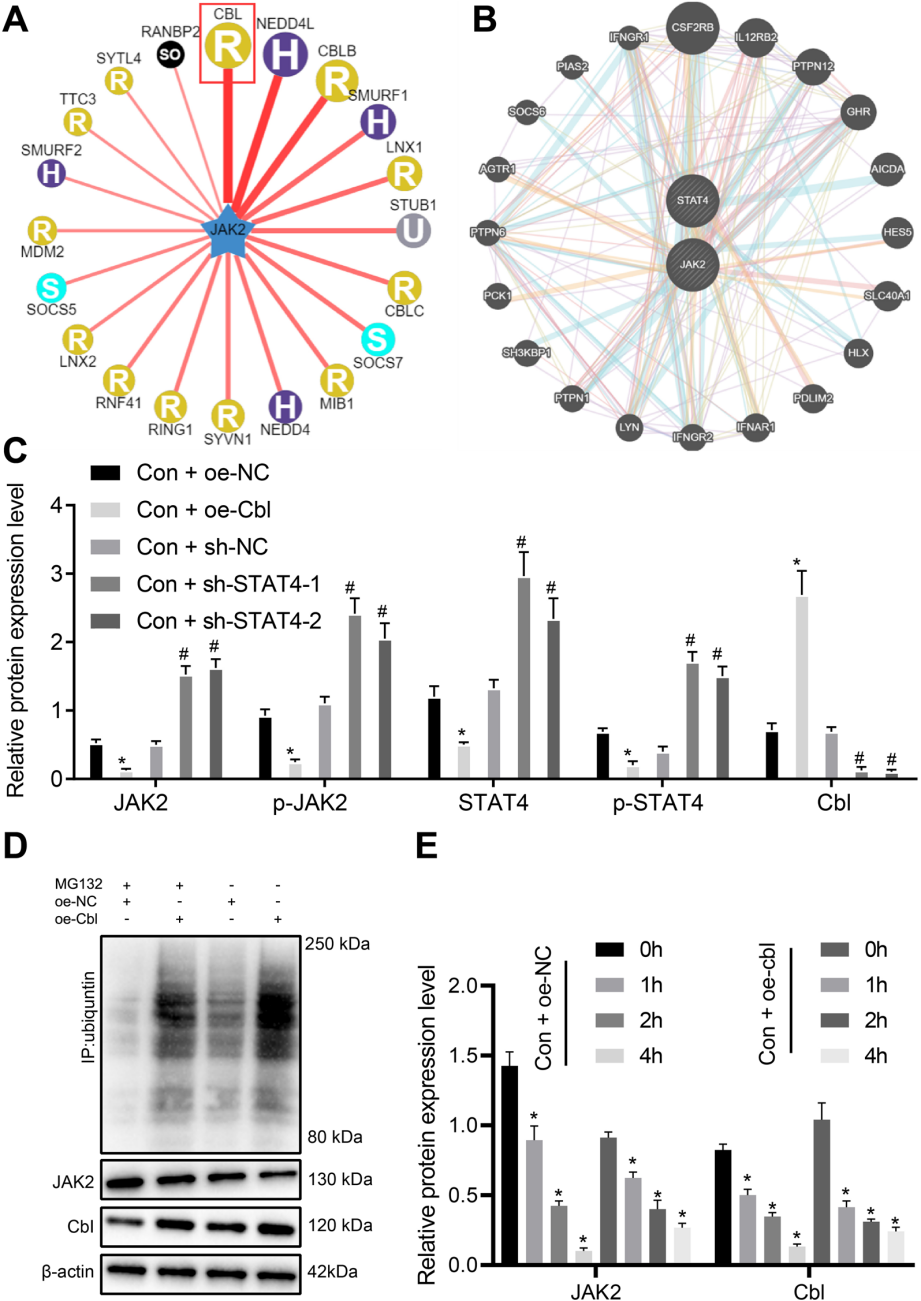
Regulation of Cbl on JAK2 ubiquitination-affected JAK2/STAT4 pathway. **A** UbiBrowser database prediction on the E3 ubiquitin ligase of JAK2. **B** PPI network of Cbl, JAK2 and related genes by GeneMANIA; the larger circle of gene indicates higher core degree, and the smaller circle of gene indicates lower core degree. **C** Western blot analysis of protein expression of Cbl, JAK2, p-JAK2, STAT4 and p-STAT4 in HUVECs cultured in normal medium upon treatment of oe-NC, oe-Cbl or sh-Cb1. * *p* < 0.05 *vs.* the Con + oe-NC group and # *p* < 0.05 *vs*. the Con + sh-NC group. **D** Western blot analysis of JAK2 ubiquitination level of HUVECs upon treatment with MG132, oe-NC, or oe-Cbl. E, Western blot analysis of JAK2 degradation level in HUVECs cells after CHX treatment. * *p* < 0.05 *vs.* 0 h. Data were measurement data, and presented as mean ± standard deviation. The data between two groups was analyzed by unpaired *t*-test. Data among groups were analyzed by one-way ANOVA, followed by Tukey’s post hoc test. Experiment was repeated three times independently

The UbiBrowser database was then used, results of which predicted that Cbl might be the E3 ubiquitin ligase of JAK2 (Fig. 3A). In addition, the PPI network of Cbl and its related genes was constructed by GeneMANIA (Fig. 3B), which proved the interaction between JAK2 and STAT4. In this work, we infected HUVECs with lentivirus harboring overexpressed/silenced Cbl to observe the alteration of JAK2 ubiquitination. Results from Western blot analysis indicated that in normal HUVECs cultured in DMEM, treatment of oe-Cbl also decreased the expressions of JAK2, p-JAK2, STAT4 and p-STAT4 (Fig. 3C) and treatment of sh-Cbl (#1, #2) exerted opposite effect, increasing JAK2, p-JAK2, STAT4 and p-STAT4. Co-IP was used to detect JAK2 ubiquitination (Fig. 3D) and JAK2 protein half-life (Fig. 3E). We found that the addition of MG-132 inhibited JAK2 ubiquitination level and upregulated JAK2 expression, and treatment of oe-Cbl elevated the JAK2 ubiquitination level in HUVECs as well as JAK2 protein degradation. The above results indicated that overexpression of Cbl inhibited the JAK2/STAT4 pathway activation by increasing JAK2 ubiquitination level and JAK2 protein degradation.

### STAT4 promotes the expression of Runx3 by regulating the H3K4me3 level in the promoter region of Runx3

**Fig. 4:**
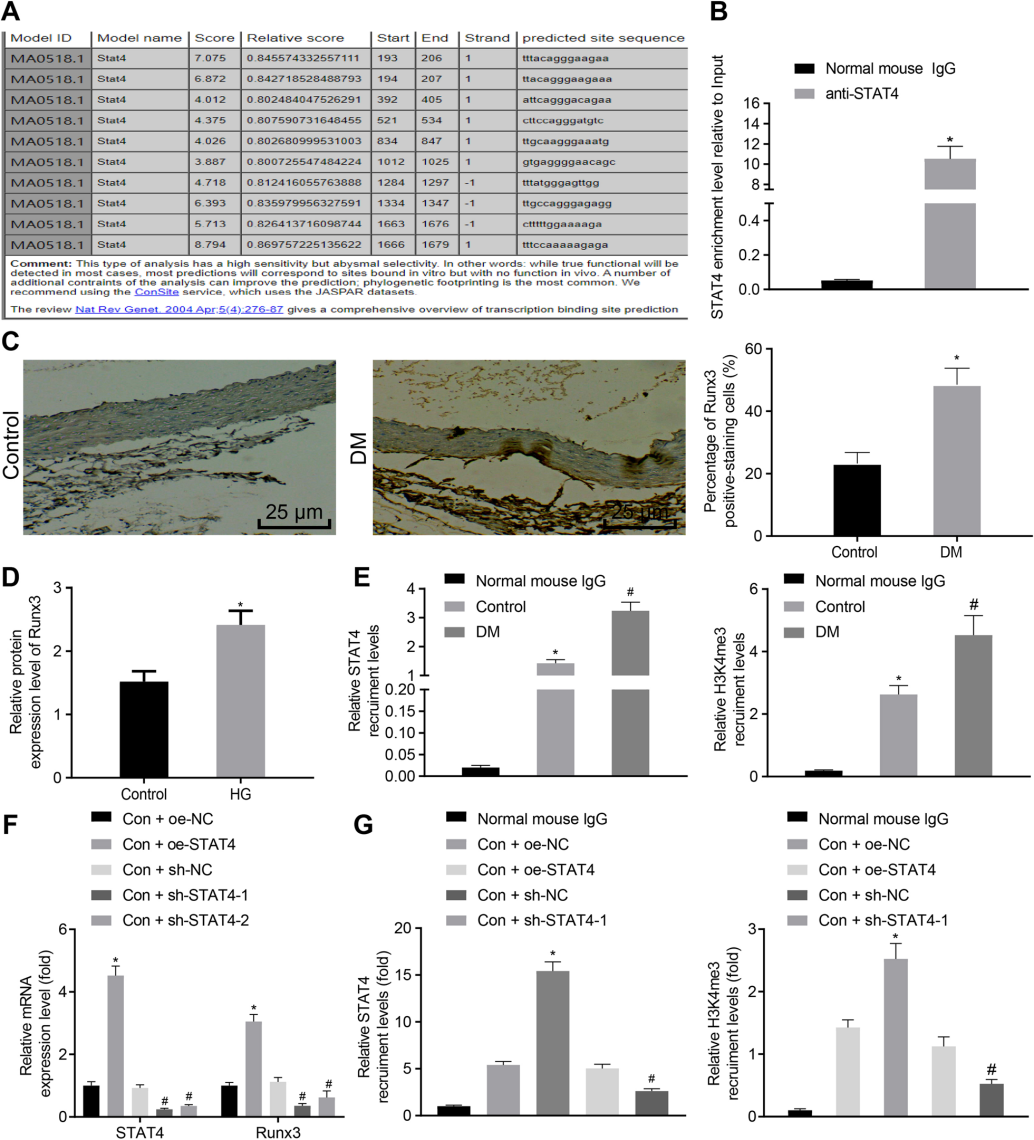
STAT4 promotes Runx3 expression by mediating the level of H3K4me3. **A** Binding site of STAT4 in Runx3 promoter region predicted by JASPAR. **B** ChIP-qPCR detected the binding of STAT4 in Runx3 promoter region. * *p* < 0.05 *vs*. the Normal IgG group. **C** IHC assay of Runx3-positive ratio in rat aortic tissues and corresponding quantification (× 400); * *p* < 0.05 *vs*. the Con group. **D** Western blot analysis of Runx3 protein expression in HUVECs under environment of Con and HG. * *p* < 0.05 *vs*. the Con group. **E** ChIP-qPCR of relative H3K4me3 and STAT4 recruitment level in the Runx3 promoter region of aortic tissues from rat aortic tissues. * *p* < 0.05 *vs*. the IgG group, # *p* < 0.05 *vs*. the Con group. **F** RT-qPCR analysis of Runx3 expression in HUVECs after treatment with Con + sh-STAT4-1, Con + sh-STAT4-2, Con + oe-STAT4 or controls. * *p* < 0.05 *vs*. the Con + oe-NC group and # *p* < 0.05 *vs*. the Con + sh-NC group. **G** ChIP-qPCR of relative H3K4me3 and STAT4 recruitment level in HUVECs under Con treatment and transfection with sh-STAT4-1, oe-STAT4 or controls. * *p* < 0.05 *vs*. the Con + oe-NC group and # *p* < 0.05 *vs*. the Con + sh-NC group. Data were measurement data, and presented as mean ± standard deviation. The data between two groups was analyzed by unpaired *t*-test. Data among groups were analyzed by one-way ANOVA. Experiment was repeated three times independently

We confirmed the presence of STAT4 binding sites in the promoter region of Runx3 through the JARSPAR database (Fig. 4A). To further indicate STAT4 binding sites in the Runx3 promoter region in HUVECs, ChIP-qPCR was conducted and revealed that STAT4 was predominantly enriched in the Runx3 promoter regions (Fig. 4B). We then performed IHC on the rat aorta from DM rats and found that Runx3 was mainly expressed in the nucleus or cytoplasm (Fig. 4C) and that the expression of Runx3 was upregulated indeed. Additionally, we also identified high Runx3 expression in HG-induced HUVECs (Fig. 4D). Then, the results from ChIP-qPCR experiment (Fig. 4E) demonstrated the increase of H3K4me3 and STAT4 recruitment levels in Runx3 promoter region of the DM aortic tissue. Similar experiments were next conducted on HUVECs. The HUVECs cultured in DMEM medium (Con) were infected with lentivirus overexpressing/silencing STAT4. The results from RT-qPCR (Fig. 4F) demonstrated that compared with oe-NC, oe-STAT4 treatment increased the expressions of STAT4 and Runx3 in HUVECs while treatment with sh-STAT4-1 as well as sh-STAT4-2 exhibited opposite effect on the STAT4 and Run3 with sh-STAT4-1 exhibiting more significantly inhibitory effect. The sh-STAT4-1 was selected for following ChIP-qPCR on HUVECs. The obtained results (Fig. 4G) revealed that oe-STAT4 treatment resulted in greater recruitment level of H3K4me3 and STAT4 in the promoter region of Runx3 while sh-STAT4-1 decreased the recruitment level of that. The above results indicated that STAT4 may bind to the Runx3 promoter region to enhance H3K4me3 levels.

### STAT4 promotes HUVEC apoptosis through Runx3

**Fig. 5:**
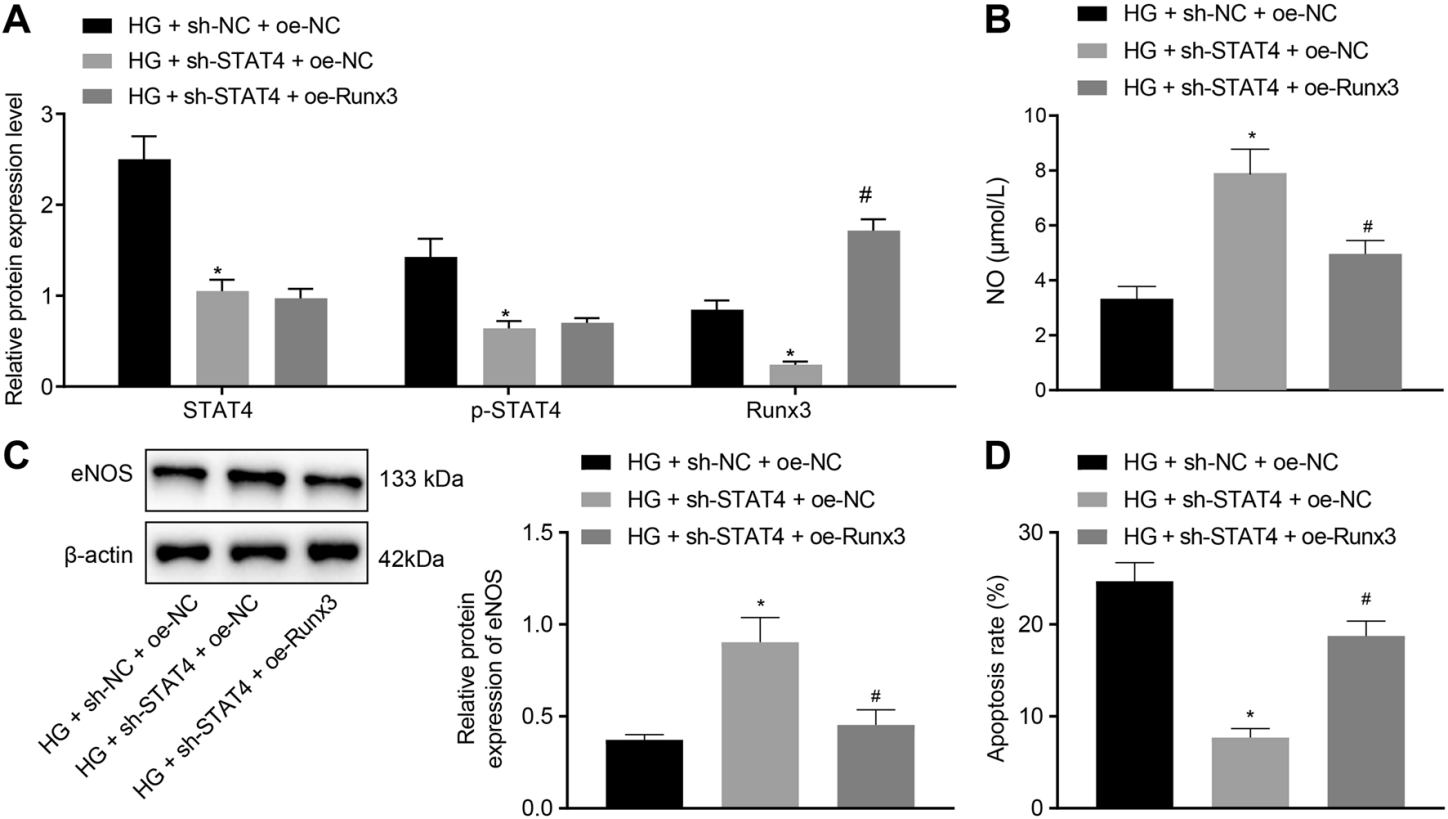
STAT4 facilitates apoptosis of HUVECs by Runx3 promotion. **A** Western blot analysis of STAT4 and Runx3 protein expression in HUVECs upon treatment with HG + sh-STAT4 + oe-NC, HG + sh-STAT4 + oe-Runx3, or controls. **B** Quantification of NO content in the culture supernatant of HUVECs upon treatment with HG + sh-STAT4 + oe-NC, HG + sh-STAT4 + oe-Runx3, or controls detected by Griess assay. **C** eNOS protein level in HG-induced HUVECs determined by Western blot analysis. **D** Flow cytometry of apoptosis of HUVECs and corresponding quantification upon treatment with HG + sh-STAT4 + oe-NC, HG + sh-STAT4 + oe-Runx3, or controls. * *p* < 0.05 *vs*. the HG + sh-NC + oe-NC group and # *p* < 0.05 *vs*. the HG + sh-STAT4 + oe-NC group. Data among groups were analyzed by one-way ANOVA. Experiment was repeated three times independently

To further indicate the specific role of STAT4 and Runx3 in DM, we exposed HUVECs to HG medium and then infected them with oe-STAT4 and sh-STAT4, followed by detection of HUVEC apoptosis. Western blot analysis indicated that silencing of STAT4 reduced STAT4 and Runx3 expression and that combined treatment of sh-STAT4 and oe-Runx3 increased Runx3 level but hardly altered STAT4 expression, compared with sh-STAT4 and oe-NC treatment in HG-induced HUVECs (Fig. 5A). NO content and eNOS protein level were detected as well and it was found that sh-STAT4 increased the NO content and eNOS protein level in HG-induced HUVECs but the additional treatment with oe-Runx3 reversed the results (Fig. 5B, C). Additionally, flow cytometry showed that treatment with sh-STAT4 resulted in decreased apoptosis of HUVECs, which was reversed by treatment with oe-Runx3 (Fig. 5D). These data suggested that interference with STAT4 expression may upregulate eNOS protein level, promote NO production and inhibit HUVEC apoptosis by inhibiting Runx3 expression.

### Cbl inhibits HUVEC apoptosis by inhibiting the activation of the JAK2/STAT4 pathway through reducing Runx3 expression

**Fig. 6:**
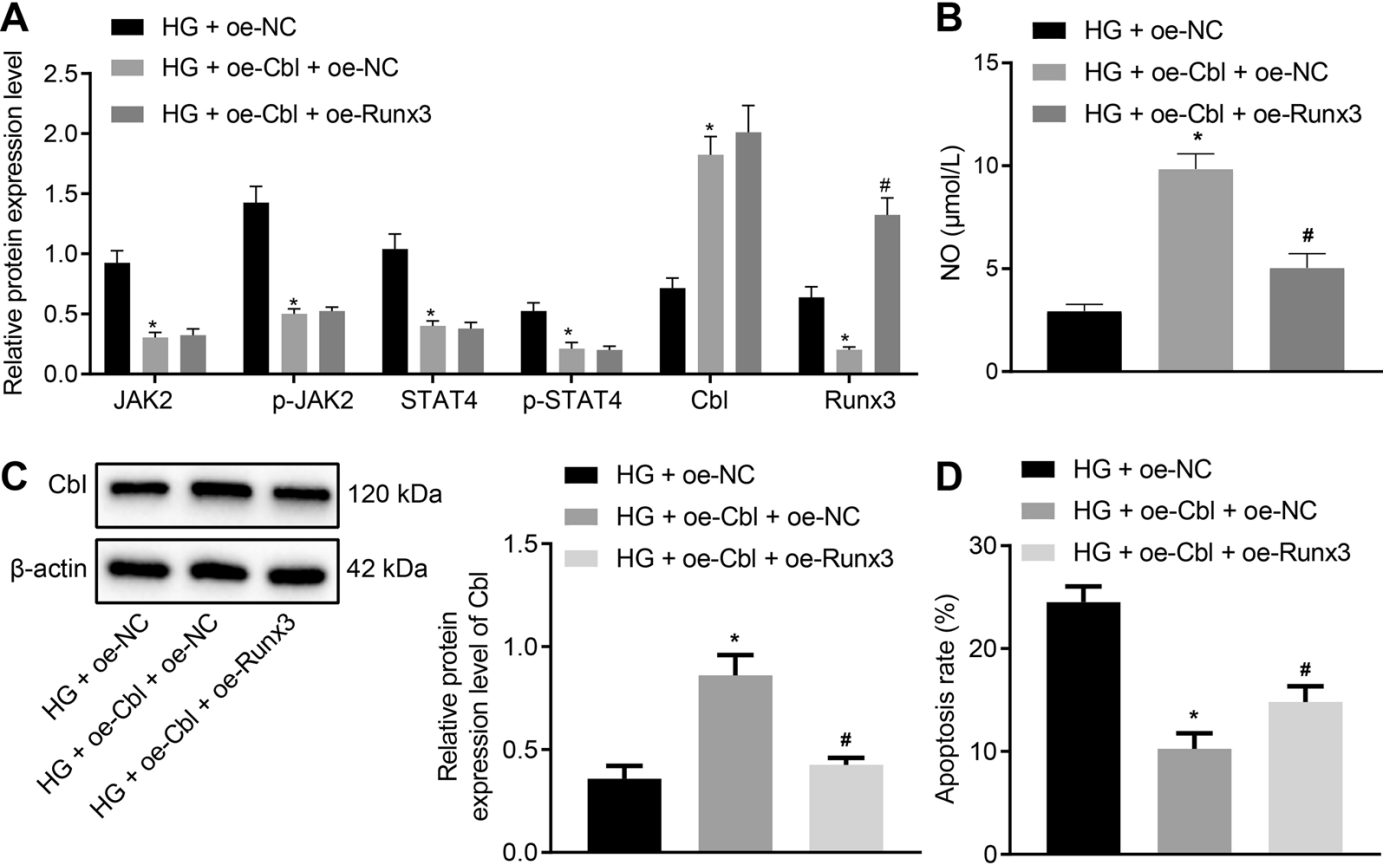
Inhibitory role of Cbl in HUVEC apoptosis through Runx3-mediated JAK2/STAT4 signaling pathway. **A** Western blot analysis of expression of Cbl, JAK2, p-JAK2, STAT4, p-STAT4 and Runx3 in HUVECs upon treatment with HG + oe-NC, HG + oe-Cb1 + oe-NC, or HG + oe-Cb1 + oe-Runx3. **B** Quantification of NO content in HUVECs upon treatment with HG + oe-NC, HG + oe-Cbl + oe-NC, or HG + oe-Cbl + oe-Runx3 using Griess method. **C** eNOS protein level in HG-induced HUVECs determined by Western blot analysis. **D** Flow cytometry of apoptosis of HUVECs and corresponding quantification upon treatment with HG + oe-NC, HG + oe-Cbl + oe-NC, or HG + oe-Cb1 + oe-Runx3. * *p* < 0.05 *vs*. the HG + oe-NC group and # *p* < 0.05 *vs*. the HG + oe-Cbl + oe-NC group. Data were measurement data, and presented as mean ± standard deviation. Data among groups were analyzed by one-way ANOVA. Experiment was repeated three times independently

Based on the above experiments, we speculated that Cbl inhibited the expression of Runx3 by inactivating the JAK2/STAT4 pathway, thereby affecting the apoptosis of HUVECs. To verify the hypothesis, HUVECs cultured with HG were further transfected with oe-Cbl and oe-Runx3, where Cbl, JAK2 and STAT4 expression was detected by Western blot analysis. It was observed that (Fig. 6A) the expression of Cbl in oe-Cbl-treated HUVECs was upregulated, and the expression of JAK2, p-JAK2, STAT4, p-STAT4 and Runx3 was downregulated. Compared with oe-Cbl + oe-NC, combined treatment with oe-Cbl and oe-Runx3 resulted in similar expression of Cbl, JAK2, p-JAK2, STAT4 and p-STAT4 (*p* > 0.05), except upregulated Runx3 expression (*p* < 0.05). As for NO content in the culture supernatant of HUVECs, we found that oe-Cbl increased NO content; based on oe-Cbl treatment, transfection for oe-Runx3 reduced the NO production (Fig. 6B). Furthermore, eNOS protein level was increased in HG-induced HUVECs overexpressing Cbl yet reduced by additional overexpressed Runx3 (Fig. 6C). Flow cytometry suggested that compared with controls, overexpressed Cbl resulted in decreased apoptosis of HUVECs but combined treatment of oe-Cbl and oe-Runx3 potentiated the capacity of apoptosis (Fig. 6D). The above results indicated that Cbl decreased the expression of Runx3 by inhibiting the activation of the JAK2/STAT4 pathway, thereby promoting the production of NO and expression of eNOS in HUVECs and inhibiting HUVEC apoptosis.

### Cbl alleviates HUVEC dysfunction in DM rats by inhibiting the activation of the JAK2/STAT4 pathway through reducing Runx3 expression

**Fig. 7:**
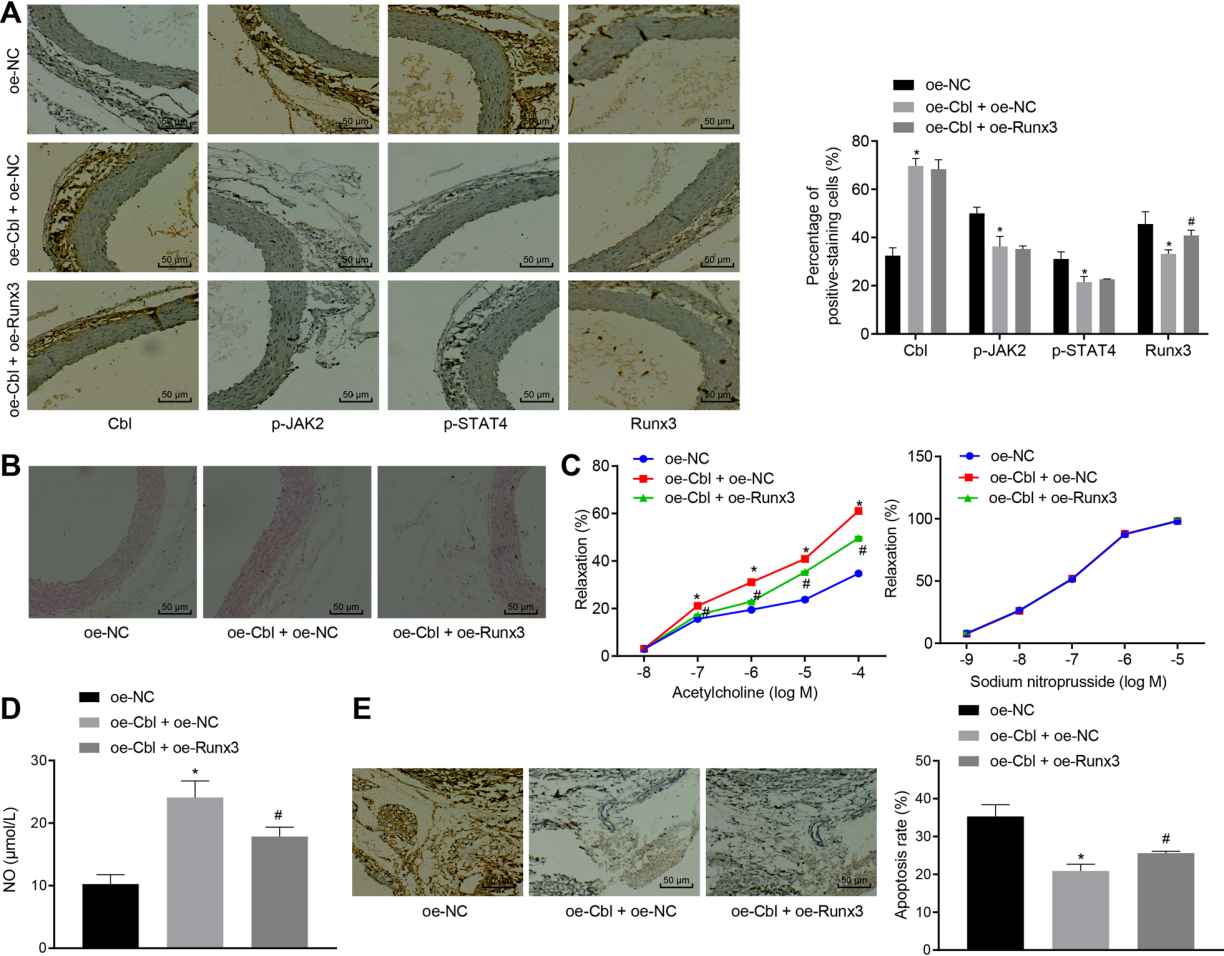
Cbl improves HUVEC dysfunction by inhibiting Runx3 expression and inactivating JAK2/STAT4 pathway. **A** IHC detecting Runx3-positive percentage in rat aortic tissues and corresponding quantification upon treatment with oe-NC, oe-Cbl + oe-NC, or oe-Cb1 + oe-Runx3 (× 200). **B** HE staining of rat aortic tissues and corresponding quantification upon treatment with oe-NC, oe-Cbl + oe-NC, or oe-Cb1 + oe-Runx3 (× 200). **C** Quantification of the aortic relaxation response to ACH and SNP in DM rats or control rats. **D** Quantification of serum NO content upon treatment with oe-NC, oe-Cbl + oe-NC, or oe-Cbl + oe-Runx3 detected by Griess assay. **E** TUNEL staining of rat aortic tissues and corresponding quantification upon treatment with oe-NC, oe-Cbl + oe-NC, or oe- + oe-Runx3 (× 200). * *p* < 0.05 *vs*. the oe-NC group and # *p* < 0.05 *vs*. the oe-Cbl + oe-NC group. Data were measurement data, and presented as mean ± standard deviation. Data among groups were analyzed by one-way ANOVA. n = 6

In vitro cell experiments above have elucidated the potential mechanism underlying Cbl inhibiting HUVEC apoptosis through regulation on the JAK2/STAT4/Runx3 axis. For comprehensive understanding on the role of Cb1 in HUVEC dysfunction in the context of DM, we further performed in vivo experiment to confirm the mechanism. Rats were injected with lentivirus expressing Cb1, Runx3 and empty vectors. Then, rats were euthanized and the aorta tissues were separated, followed by functional assays. IHC initially indicated that (Fig. 7A) oe-Cbl treatment effectively enhanced the expression of Cbl in the aorta tissues while the expression of p-JAK2, p-STAT4 and Runx3 was all downregulated; addition of oe-Runx3 did not further alter expression of Cbl, p-JAK2 and p-STAT4 in the aortic tissues (*p* > 0.05) but only increased Runx3 expression. The results of HE staining (Fig. 7B) demonstrated that upon treatment with either oe-NC or oe-Cbl + oe-Runx3, the aorta intima lost integrity, volume of endothelial cells increased, partial endothelial cells shed, smooth muscle cells of tunica media arranged disorderly, vacuole degenerated, and thickness of elastic plate varied. However, upregulation of Cb1 significantly attenuated these symptoms and even cells of each layer tended to be regularly arranged, indicating that Cbl reduced the aortic tissue lesions in DM rats. In addition, in oe-Cb1-treated aorta rings, endothelial-dependent relaxation ratio increased secondary to the increasing ACH concentration in a dose-dependent manner, more effective than that in oe-NC-treated aorta rings, but the combined treatment of oe-Cb1 and oe-Runx3 alleviated the dependence on ACH concentration. Meanwhile, increasing SNP hardly affected blood vessel relaxation upon whichever treatments (*p* > 0.05) (Fig. 7C). We next detected NO content in rat serum upon treatments. Compared with that of the oe-NC-treated rats, the serum NO content of the oe-Cbl + oe-NC-treated rats increased while the treatment of oe-Runx3 decreased the increase induced by oe-Cbl (Fig. 7D). To confirm the impact of Cbl and Runx3 on apoptosis and we performed TUNEL staining (Fig. 7E). It was clear that overexpression of Cbl decreased the apoptosis of aortic tissues, while addition of oe-Runx3 increased the apoptotic ratio. Taken altogether, Cbl inhibited the expression of Runx3 by inhibiting the activation of the JAK2/STAT4 pathway, and activated NO production, ultimately inhibiting HUVEC apoptosis in the aorta of DM rats.

## Discussion

Endothelial dysfunction is characterized by impaired endothelium-dependent vasodilation and endothelial activation, associated with insulin-resistance, inflammation and hyperglycemia, while it appears to precede progression of DM [25]. Vascular endothelial cells normally perform several key homeostatic functions, but upon injury or cell death, dysfunction of endothelial cells predisposes diabetic patients to cardiovascular complications and microthrombus formation [26]. Therefore, endothelial dysfunction is indicated as a potential biomarker for DM treatment and the mechanism of it deserves more comprehensive investigation. Herein, our work elucidated that E3 ubiquitin ligase Cbl stimulates NO production and inhibits apoptosis of HUVECs, thereby improving endothelial function in DM rats.

NO is known to play crucial roles in the regulation of blood flow through vasodilatation and decreased vascular resistance in various organs and tissues; NO derived from eNOS also contributes to the inhibition of platelet aggregation, adhesion, and smooth muscle proliferation [27]. But in DM, NO bioavailability is impaired by the increased free radicals production, and the reactive oxygen species oxidize the cofactors of the NOS, consequently leading to a decreased NO production [28]. Improvement of NO bioavailability and production thus becomes one of promising therapeutic strategies. Researchers have explored the potential of a transdermal NO application for the treatment of diabetic wound ulcers by increasing vasodilation [29]. In this study, injection of oe-Cbl resulted in the increased NO content in rat serum and HUVECs under environment of HG, as Cbl was poorly expressed in DM. The Cbl-b regulates T cell activation thresholds and contributes to immune response against DM; Cbl-b deficiency predisposes to diabetes and formation of islet autoantibodies in mice [14]. Cbl-b negatively regulated nuclear factor-kappa B (NF-κB) in T-cells and the loss of Cbl-b results in aberrant activation of NF-κB upon T-cell antigen receptor ligation [30]. Long-term activation of the innate immune system impairs insulin secretion and action, and inflammation also contributes to macrovascular and microvascular complications of diabetes [31]. We observed that in DM rats, the aorta intima lost integrity, volume of endothelial cells increased, and partial endothelial cells shed. Upregulation of Cbl significantly attenuated these symptoms. Endothelial cells exposed to hyperglycemia face an apoptotic process, leading to intimal denudation, and the downregulation of vascular endothelial-cadherin results in endothelial apoptosis that takes place mainly at arterial sites [32].

Mechanistically, we found that Cbl inhibits the JAK2/STAT4 signaling pathway by elevating ubiquitylation of JAK2, thereby inducing NO production and suppressing apoptosis of HUVECs. HG conditions facilitated the recruitment of STAT4 [17] and STAT4 is overexpressed in peripheral blood mononuclear cell from patients with DM, especially with more severe reaction against β-cell antigens [33]. In the only the early-onset cases of autoimmune DM, STAT4 alleles and haplotype might influence cytokine signaling and promotes progression of the disorder [34]. In the present study, we provided experimental data demonstrating that overexpression of STAT4 or JAK2 decreased NO production and increased apoptosis of HUVECs, abrogating the effect of Cbl. JAK2 activation is crucial for cytokine receptor signal transduction and leukemogenesis, while Cbl specifically promotes JAK2 ubiquitination and following granulocyte–macrophage colony stimulating factor (GM-CSF) stimulation, the levels of p-JAK2 are reduced which is rescued by Cbl expression [18]. Antisense inhibition of c-Cbl expression led to enhanced JAK-STAT activation following GM-CSF stimulation, associated with kinase activity and DNA synthesis [35].

JAK2/STAT pathway previously has been implicated in immune system and apoptosis. The JAK2/STAT3/SOCS axis contributes to the development of DM by mediating inflammation associated with vascular endothelial cells and/or monocytes [36]. Cryptotanshinone, fat-soluble phenanthrene quinone, inhibits cell progression of lung tumors by increasing CD4^+^ T cell toxicity through activation of the JAK2/STAT4 pathway [37]. Tribulus terrestris, an annual herb, is indicated to suppress HUVEC proliferation and apoptosis rates, while it prolongs the HUVEC survival time and postpones the decaying stage also through inactivation of the JAK2/STAT3 pathway [38]. In a word, downregulation of JAK2 and STAT4 might alleviate endothelial dysfunction in DM rats. Similarly, treatment with a specific JAK1/2 inhibitor for 2 weeks partly reversed the major phenotypic changes of diabetic kidney disease [39]. Disruption of STAT4 activation completely prevents the development of spontaneous diabetes in mice [40]. Moreover, we indicated that silencing of STAT4 stimulated the production of NO in HUVECs and inhibited apoptosis, which was reversed by overexpression of Runx3; moreover, the overexpressed Runx3 alleviated the inhibitory effect of Cbl on apoptosis of HUVECs. Knockdown of Runx3 expression alleviates EPC dysfunction in diabetic patients as well as maintains autophagy of HUVECs [20, 41]. STAT4-mediated epigenetic control of individual Runx3 transcription factors promotes the adaptive behavior of antiviral natural killer cells which are innate lymphocytes, as Runx1 and Runx3 are targets of STAT4 [19].

## Conclusion

**Fig. 8:**
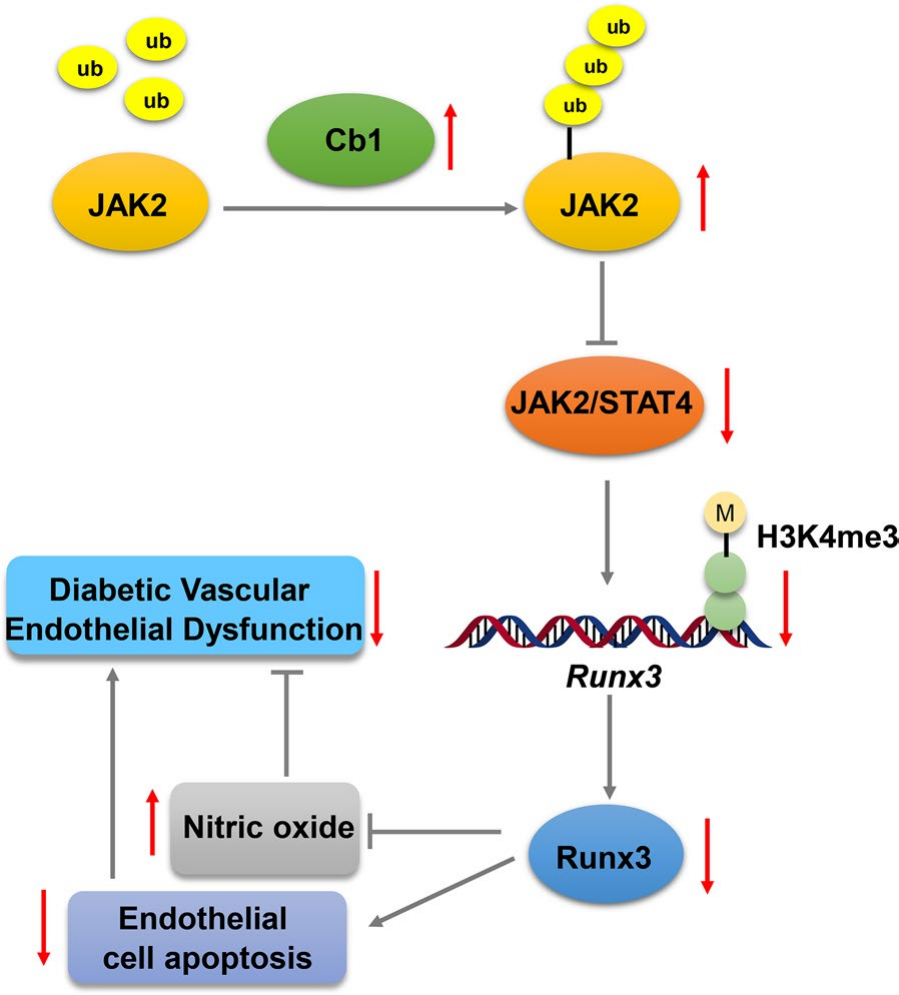
The mechanistic diagram depicting the role of Cbl in HUVEC dysfunction in DM. Cbl blocked the JAK2/STAT4 pathway to downregulate the expression of Runx3 through H3K4me3 modification, thereby alleviating HUVEC dysfunction in DM

In conclusion, this study elucidates the mechanism that Cbl alleviates endothelial dysfunction in DM through inhibiting the JAK2/STAT4 pathway and Runx3 expression (Fig. 8), providing novel insight into targeted therapy against DM and comprehensive understanding on DM progression.

## Supplementary Information

**Additional file 1: Table S1**. Primer sequence for RT-qPCR. **Table S2**. Primer sequence for ChIP-qPCR.

## Data Availability

The datasets generated and/or analysed during the current study are available from the corresponding author on reasonable request.

## Abbreviations

DM: Diabetes mellitus
NO: Nitric oxide
EPCs: Endothelial progenitor cells
Cbl: Casitas B-lineage lymphoma
HUVECs: Human umbilical vein endothelial cells
STAT4: Signal transducer and activator of transcription 4
