# Supplementary material for: Overexpression of E3 ubiquitin ligase Cbl attenuates endothelial dysfunction in diabetes mellitus by inhibiting the JAK2/STAT4 signaling and Runx3-mediated H3K4me3
Source: J Transl Med. 2021 Nov 19;19:469. doi: 10.1186/s12967-021-03069-w (PMC8605525; doi:10.1186/s12967-021-03069-w)
Supplement: Supplementary file 1 — Additional file 1: Table S1. Primer sequence for RT-qPCR. Table S2. Primer sequence for ChIP-qPCR. [file 12967_2021_3069_MOESM1_ESM.docx]

**Table S1** Primer sequence for RT-qPCR

| Species | Gene | Sequence |
| --- | --- | --- |
| Human | STAT4 | F: 5'-TGTTGGCCCAATGGATTGAAA-3' |
|  |  | R: 5'-GGAAACACGACCTAACTGTTCAT-3' |
|  | Runx3 | F: 5'-AGGCAATGACGAGAACTACTCC-3' |
|  |  | R: 5'-CGAAGGTCGTTGAACCTGG-3' |
|  | β-actin | F: 5'-GTGACGTTGACATCCGTAAAGA-3' |
|  |  | R: 5'-GCCGGACTCATCGTACTCC-3' |

Note: RT-qPCR, reverse transcription quantitative polymerase chain reaction; F, forward; R, reverse; STAT4, signal transducer and activator of transcription 4; Runx3, runt-related transcription factor 3.

**Table S2** Primer sequence for ChIP-qPCR

| Gene | Sequence |
| --- | --- |
| Runx3 (Human) | F: 5'-GTTCCGTTTTGGATGCGCCCTGCA-3' |
|  | R: 5'-CAAAACCCCATCCGCCCATTTCCGCA-3' |
| Runx3 (Rat) | F: 5’-ATCCACTTCCACTACACCGG-3’ |
|  | R: 5’-ACGGTGTGCATTTTGGAGTC-3’ |

Note: ChIP-qPCR, chromatin immunoprecipitation-quantitative polymerase chain reaction; F, forward; R, reverse; Runx3, runt-related transcription factor 3.
